# Validation of magnetic resonance relaxometry R2 value and cyst fluid iron level for diagnosis of ovarian endometrioma

**DOI:** 10.1080/13510002.2021.1937456

**Published:** 2021-06-04

**Authors:** Shogo Imanaka, Yuki Yamada, Naoki Kawahara, Hiroshi Kobayashi

**Affiliations:** a Department of Obstetrics and Gynecology, Nara Medical University, Kashihara, Japan; b Ms.Clinic MayOne, Kashihara, Japan

**Keywords:** Iron, magnetic resonance relaxometry, endometriosis, ovarian cancer, ovarian endometrioma, antioxidants, bioelements, diagnosis

## Abstract

**Objectives:**

Magnetic resonance (MR) R2 relaxometry is a safe, noninvasive diagnostic modality for the evaluation of iron levels in the contents of ovarian cysts. The study aims to investigate the sensitivity and specificity of the two methods, R2 value and iron level, in diagnosing OMA patients in the validation set.

**Methods:**

A prospective cohort study was conducted from 2013 to 2019. We investigated how R2 value was affected by iron-related compounds, antioxidants and bioelements in the cysts.

**Results:**

The sensitivity and specificity of CF iron-based diagnosis of OMA was 96.6% and 95.4%, respectively. The sensitivity and specificity for R2 value in diagnosing OMA were 86.2% and 70.7%, respectively. The outcomes of the two tests were highly correlated (r = 0.758; P <0.001). The R2 value was positively correlated with CF levels of iron-related compounds and antioxidants. The R2 value was affected not only by iron ions but also by calcium ions.

**Conclusion:**

Preoperative MR relaxometry may provide a noninvasive alternative to CF iron test in diagnosing OMA. The presence of paramagnetic cations in the cyst may be associated with reduced specificity.

## Introduction

Endometriosis is an estrogen-dependent chronic inflammatory disease that affects around 5% of reproductive age women [1]. Endometriosis is a common cause of dysmenorrhea and infertility and is sometimes associated with ovarian cancer [1]. Endometriosis is commonly associated with inflammation and oxidative stress [2]. Yamaguchi et al. first reported in 2008 that the contents of the endometriotic cysts were rich in free iron and under excessive oxidative stress [3]. Elevated levels of reactive oxygen species (ROS) can stimulate the growth of endometriotic lesions [4]. This suggests that iron overload and iron-catalyzed oxidative stress may be associated with the pathogenesis of endometriosis [5]. Recent animal models of endometriosis have successfully linked iron deposition and periovarian fibrosis to oxidative stress as evaluated by 4-hydroxy-2-nonenal-modified proteins and 8-hydroxy-2′-deoxyguanosine (8-OHdG) [6]. It has also been reported that a redox imbalance between ROS and antioxidants due to repetitive bleeding is involved in ovarian carcinogenesis [5]. Cyst fluid samples were collected during surgical procedures in patients with OMA and EAOC and analyzed for iron concentration. Yoshimoto et al. reported in 2015 that iron levels in ovarian cysts are useful markers for differentiating between OMA and ovarian cancer, especially EAOC [7]. In the training cohort, the cyst fluid (CF) total iron exhibited a sensitivity of 85% and specificity of 98% with cut-off value of 64.8 mg/L for discriminating OMA from non-OMA [7]. These studies demonstrated that accurate quantification of cyst fluid iron concentration can distinguish EAOC from OMA. However, current iron measurement approaches require surgery and are invasive.

Recent advances in innovative imaging technology have enabled non-invasive quantification of iron. There are different MR techniques and models to quantify iron concentration in human organs such as liver and heart [8]. We have focused on MR relaxometry for measuring iron levels [9]. MR relaxometry R2 values correlated with iron levels, indicating that CF iron levels were significantly higher in OMA than in EAOC [9]. To distinguish OMA from EAOC, the cut-off R2 value of 12.1 s^−1^ had a sensitivity of 86% and a specificity of 94% [9]. Thus, noninvasive MR relaxometry enables the measurement of iron-related compounds with high sensitivity and high specificity.

Ovarian masses may be benign, borderline, or malignant. Benign ovarian tumors include endometriosis and non-endometriotic ovarian cysts. Furthermore, ovarian cancer comprises a heterogeneous group of tumors, including serous, clear cell, endometrioid, and mucinous carcinoma. This study quantified MR relaxometry R2 value in patients with all types of ovarian masses. The study aims to evaluate the usefulness of these cut-off values in diagnosing OMA using the validation dataset. In addition to iron-related compounds, we also investigate molecules and bio-elements that may affect the R2 values.

## Materials and methods

### Patient selection

A single-center prospective cohort study was conducted by collecting clinical data and cyst fluid samples from patients admitted to the Department of Gynecology, Nara Medical University Hospital, Kashihara, Japan, from January 2013 to May 2019. Participants underwent transvaginal ultrasonography (TVS) for the diagnosis of ovarian cysts, followed by magnetic resonance imaging (MRI) and MR relaxometry. The inclusion criteria were: (1) patients who have undergone TVS, MRI, and MR relaxometry; (2) who have undergone surgery; and (3) who have collected cyst fluid. The criteria for exclusion were: (1) age below 20 years; (2) women during menstruation; (3) antioxidant supplement use; (4) hormonal therapy within 3 months; and (5) chemotherapy before surgery. The total iron cutoff value (64.8 mg/L) that distinguishes between OMA and non-OMA was reported in the training set [7]. The cutoff value for MR relaxometry R2 value is 12.1 s^−1^, which was also determined in the training set [2]. This study includes 307 histopathologically confirmed cases (178 cases of OMA, 41 cases of EAOC [clear cell carcinoma, *n* = 20, endometrioid carcinoma, *n* = 13, and others, *n* = 8], 57 cases of non-OMA benign ovarian cysts [serous adenoma, *n* = 19; mucinous adenoma, *n* = 18; mature teratoma, *n* = 10; and others, *n* = 10] and 31 cases of non-EAOC [high grade serous carcinoma, *n* = 24 and others, *n* = 7]) as the validation set. Clinicopathological data such as general demographic characteristics, patient history, laboratory values, medications, and pathology were collected from an electronic database containing comprehensive medical records and pathology reports. The study was conducted under the guidelines that had been approved by the Medical Ethics committee of the Nara Medical University (reference no. 2012–541 and 2012-951). Written informed consent was obtained from each patient. The treatment and care of these patients complies with the Guideline for Gynecological Practice in Japan established by the Japan Society of Obstetrics and Gynecology and Japan Association of Obstetricians and Gynecologists [10].

## MR relaxometry for determining R2 value

All patients underwent routine MRI using T1W and T2W sequences. MRI were obtained on a 3 T system (Magnetom Verio, Siemens Healthcare, Erlangen, Germany). After the routine clinical MRI, participants underwent MR relaxometry by using single-voxel acquisition mode sequence at multiple echo times and by fitting an exponential decay to the echo amplitude at different multiple echo times [9]. A parameter R2 value (s^−1^) was calculated using high-speed T^∗^2 -corrected multiecho MR sequence (HISTO) by the 3T-MR system that has been described previously [9].

## Cyst fluid collection

To evaluate the effectiveness of the two independent diagnostic algorithms, the collected samples from the ongoing project were used as the validation set. Cyst fluid samples were collected from the study population at the time of surgery. The CF was centrifuged at 3000 g for 5 min; the cell-free fluid was stored at −80°C for future analysis. Biomarkers (iron-related compounds, antioxidants, and cations) were quantified using the cyst fluid samples of registered cases.

## Measurement of total iron, heme iron, and free iron concentration

Frozen-thawed cyst fluid was diluted and added to a 96-well spectrophotometric plate, and then alkalized with NaOH to adjust the pH to >10. The resultant solution was subjected to total iron, heme iron, and free iron measurements using the Metalloassay LS Assay kit for total iron, heme iron, and free iron (Metallogenics Co., Ltd.), based on the Triton-methanol colorimetric assay [7]. The optical density at 400 nm was determined using a microtiter plate reader.

## Measurement of the electronic absorption spectra of oxyHb and metHb

The electronic absorption spectra of different species of hemoglobin in cyst fluid were investigated as described in ref. [11]. Cyst fluids were added to 96-well plates and the electronic absorption spectra of the samples were measured with a grating spectrophotometric microplate reader. A peak at 620 nm in the spectrum indicates a specific absorption of metHb, whereas a peak located at 580 nm represents the absorption spectrum of oxyHb [11].

## Measurement of 8-OHdG, HO-1, bilirubin, and TAC

Cyst fluid samples were used for the measurement of 8-OHdG levels using a competitive in vitro ELISA kit (Catalog# KOG-HS10/EC, NIKKEN SEIL Co., Ltd, Shizuoka, Japan), heme oxygenase-1 (HO-1) levels using StressXpress® HO-1 ELISA kits (Catalog# SKT-111-96, StressMarq Biosciences, Inc., Victoria, BC, Canada), bilirubin levels using colorimetric ELISA kits (Redox assay, MG Metallogenics, Japan) and total antioxidant capacity (TAC) using the TAC Assay kit (Metallogenics Co., Ltd., Chiba, Japan) as described [12]. Dilution linearity and parallelism, in addition to intra- and inter-assay variability, were assessed using in-house calibrators. The ELISA kits exhibited a linear response in the range of ≥3 orders of magnitude. The assay variances of all methods described above were <10%. Each assay was performed in duplicate or triplicate.

## Measurements of the concentration of bioelements (cations): Ca, Cu, Fe, Mg, and Zn in cyst fluid samples

Cyst fluid sample was diluted with distilled water. Contents of bioelements (cations) were established by means of atomic absorption spectrometry in an oxygen acetylene flame using the working wavelength for Ca (396.8 nm), Cu (324.8 nm), Fe (259.9 nm), Mg (279.6 nm), and Zn (213.9 nm) [13]. The cation concentration was calculated as mg/L.

## Statistical analysis

SPSS 25.0 (SPSS Inc., Chicago, IL) statistical software was used for the statistical analysis. Student’s t-test was used for the analysis of continuous variables. Comparison of categorical variables between groups was performed using the Chi-square test. Data distribution was verified by Shapiro–Wilk test. Data on all types of iron, antioxidants, and bio-elements showed a non-normal distribution. The Kruskal–Wallis test followed by Bonferroni correction was performed for comparing nonnormally distributed multiple variables. Correlations between the variables were determined employing Spearman correlation. The level of significance was set at 0.05 for all tests.

## Results

### Demographics and baseline characteristics of the study cohort

Clinical characteristics of the participants are presented in Table 1. Age at diagnosis (*P* < 0.001) and the maximum diameter of the cyst (*P* < 0.001) differed significantly between the four groups. Age at diagnosis was significantly different among the four groups, with OMA patients being the youngest (Kruskal–Wallis test followed by Bonferroni's correction, *P* < 0.001). Tumor size was significantly different among the four groups, with OMA patients being the smallest (*P* < 0.001). There were no statistical differences in parity (*P* = 0.294) and CA125 levels (*P* = 0.720) between the four groups.

**Table 1.** Demographics and baseline characteristics of the study cohort.

Baseline characteristics of four groups	OMA *n* = 178		EAOC *n* = 41		Non-OMA *n* = 57		Non-EAOC *n* = 31		*P*-value
Age at diagnosis, y*	38.1	8.775	51.2	10.897	43.4	16.729	46.3	14.523	<0.001 ^1)^
Parity, n**	0	0–3	1	0–3	0	0–3	1	0–3	0.294 ^2)^
Tumor size, mm**	60	10–193	140	42–225	72	29–301	104	30–255	<0.001 ^3)^
CA125 U/ml**	61.0	7.0–1504.0	188.0	8.0–6272.0	15.0	4.0–108.0	25.0	7.0–871.0	0.720 ^3)^

### CF total iron levels and R2 value for each group

**Figure 1.**
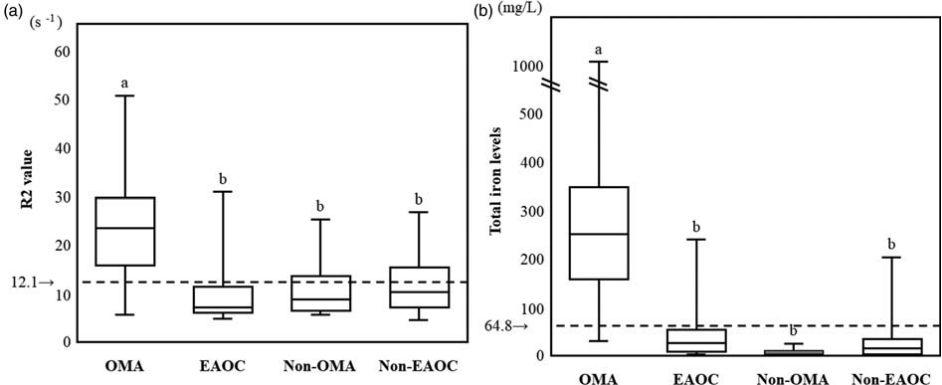
Cyst fluid R2 values and total iron levels among the four groups. This figure shows the distribution of R2 values (A) and total iron levels (B) for each studied group. a vs. b, *P* < 0.001 by the Kruskal-Wallis test followed by Bonferroni's correction.

Figure 1 shows the box and whisker plots representing median levels and the interquartile range (box) of R2 values (Figure 1(A)) and total iron (Figure 1(B)) for each studied group. ‘12.1 s^−1^’ and ‘64.8 mg/L’ indicate the previously determined R2 and total iron cutoff values to distinguish between OMA and non-OMA in the training set, respectively [7,8]. R2 values were significantly different among the four groups, with OMA patients having the highest values (Kruskal–Wallis test followed by Bonferroni's correction, *P* < 0.001) (Table 2 and Figure 1(A)). The characteristics and validity of the R2 test and CF iron test were reflected by the sensitivity and specificity. R2 values in OMA patients were significantly higher than those in the other three groups (non-OMA patients). With a cutoff value of 12.1 s^−1^ obtained from the training set, the sensitivity and specificity for distinguishing OMA from non-OMA patients were 86.2% (95% confidence interval [95% CI], 70.1–93.8%) and 70.7 (95% CI, 49.8–81.9%), respectively, in the validation set.

**Table 2.** CF total iron levels and R2 value for each group.

	OMA *n* = 178		EAOC *n* = 41		Non-OMA *n* = 57		Non-EAOC *n* = 31		*P*-value
R2, s^−1^	22.800 ^a^	6.730–51.770	7.600 ^b^	4.800–31.220	9.025 ^b^	5.960–25.320	10.335 ^b^	4.600–27.400	<0.001*
Total iron, mg/L	247.016 ^c^	27.706–1046.280	20.119 ^d^	0.888–237.619	0.865 ^d^	0.000–27.293	11.051 ^d^	0.437–201.730	<0.001*

In a parallel study, CF total iron levels were significantly different among the four groups, with OMA patients having the highest values (Kruskal–Wallis test followed by Bonferroni's correction, *P* < 0.001) (Table 2 and Figure 1(B)). CF total iron levels in OMA patients were significantly higher than in non-OMA patients. CF total iron value achieved the sensitivity of 96.6% (50% CI, 90.5–99.8%) and the specificity of 95.4% (50% CI, 89.9–98.9%) in distinguishing between OMA and non-OMA patients.

### Correlation of R2 values with iron-related compounds and antioxidants in the cysts

**Figure 2.**
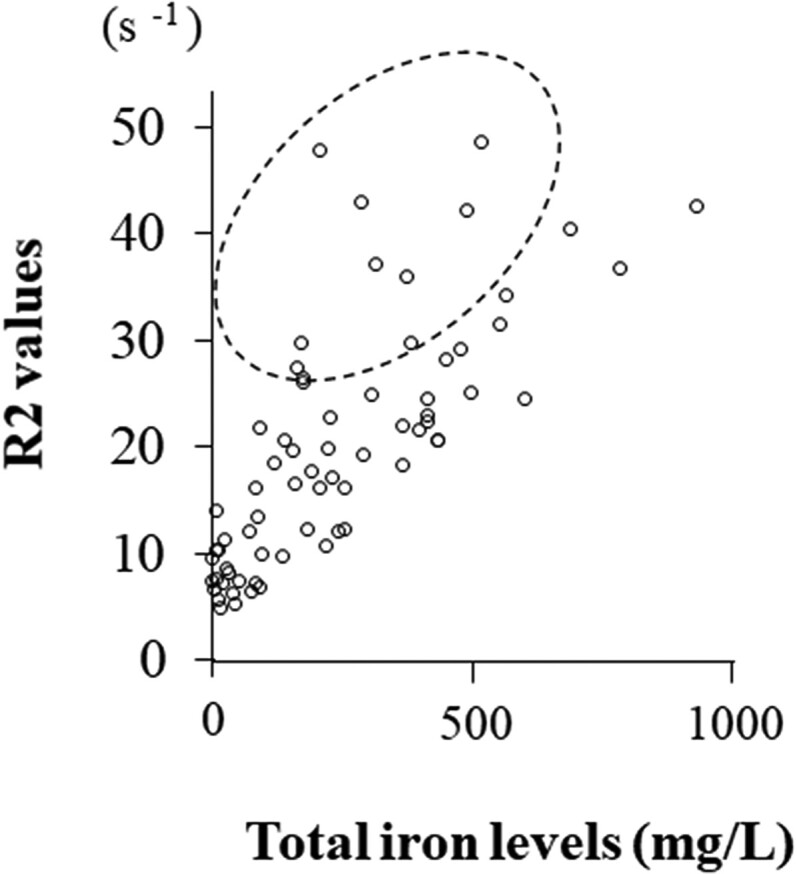
Correlation between cyst fluid total iron concentrations and R2 values. Patients in the area surrounded by the dotted line showed higher R2 values than expected from total iron levels.

First, we examined whether all types of iron-related compounds correlate with oxidative marker (8-OHdG) and all antioxidant markers (bilirubin, HO-1, and TAC) in all study cohorts. As shown in Table 3, all types of iron-related compounds except oxyHb and metHb were significantly positively correlated with all antioxidant markers (bilirubin, HO-1 and TAC). Only free iron correlated with 8-OHdG. We next investigated the correlation of R2 values with cyst fluid oxidants (Total iron, heme iron, free iron, OxyHb, MetHb, 8-OHdG) and antioxidants (Bilirubin, HO-1, TAC) in all study cohorts. Table 4 showed that R2 values were correlated with total iron (*P* < 0.001), heme iron (*P* < 0.001), free iron (*P* < 0.001), HO-1 (*P* < 0.001), and TAC (*P* = 0.008), but not with oxyHb (*P* = 0.195), metHb (*P* = 0.096), 8-OHdG (*P* = 0.369), or bilirubin (*P* = 0.414). R2 values showed higher correlation coefficients in the order of total iron, free iron, HO-1, heme iron, and TAC. The R2 value was best correlated with total iron (*r* = 0.758, *P* < 0.001), but in some populations the actual R2 value was higher than those expected from total iron (Figure 2).

**Table 3.** Correlation of all types of iron-related compounds with oxidative marker (8-OHdG) and all antioxidant markers (bilirubin, HO-1, and TAC) in the cyst.

	8-OHdG		Bilirubin		HO-1		TAC	
	*r*	*p*	*r*	*p*	*r*	*p*	*r*	*p*
Total iron	0.226	0.083	**0**.**389**	**0**.**004**	**0**.**636**	**<0.001**	**0**.**655**	**<0.001**
heme iron	0.072	0.592	**0**.**577**	**<0.001**	**0**.**484**	**<0.001**	**0**.**348**	**0**.**004**
free iron	**0**.**554**	**<0.001**	**0**.**333**	**0**.**024**	**0**.**398**	**0**.**001**	**0**.**554**	**<0.001**
OxyHb	−0.119	0.441	−0.010	0.941	−0.151	0.290	0.022	0.880
MetHb	−0.116	0.454	−0.010	0.945	−0.137	0.338	−0.008	0.955

**Table 4.** Correlation of R2 values with iron-related compounds and antioxidants in the cysts.

	Total iron		heme iron		free iron		OxyHb		MetHb		8-OHdG		Bilirubin		HO-1		TAC	
	*r*	*p*	r	*p*	*r*	*p*	*R*	*p*	*r*	*p*	*r*	*p*	*r*	*p*	*r*	*p*	*r*	*p*
R2, s^−1^	0.758	<0.001	0.520	<0.001	0.655	<0.001	0.173	0.195	0.221	0.096	0.162	0.369	−0.237	0.414	0.575	<0.001	0.422	0.008

### Correlation of R2 values with bioelements (cations) in the cysts

**Figure 3.**
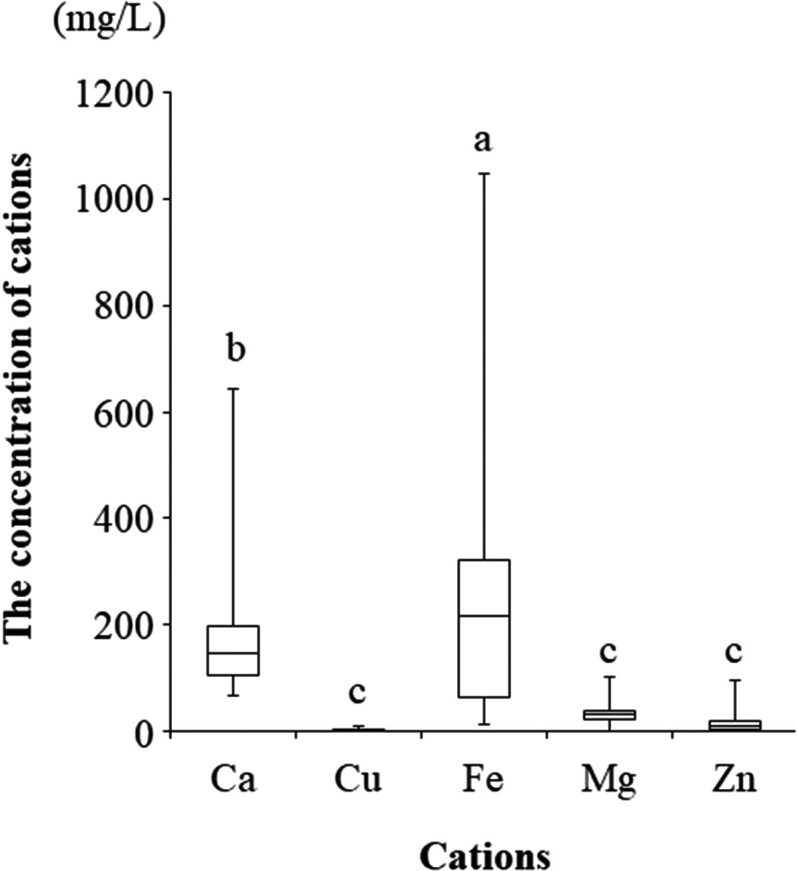
Concentration of bio-elements (cations) in the contents of ovarian cysts. a vs. b, *P* < 0.05; b vs. c, *P* < 0.001; and a vs. c; *P* < 0.001 by the Kruskal-Wallis test followed by Bonferroni's correction.

Since the relaxation time T2 is known to be affected by calcium ion concentration [14], the concentration of bioelements (cations) in the cyst fluid was measured. As shown in Figure 3, iron and calcium ions in the cyst fluid were significantly higher than other cations. There was a strong positive correlation between calcium ions and iron ions (*r* = 0.912; *P* < 0.001; data not shown). We investigate whether the R2 value is correlated with cations in the cysts. Table 5 showed that R2 values were correlated with Fe (*P* = 0.001) and Ca (*P* = 0.016), but not with Cu (*P* = 0.110), Mg (*P* = 0.418), or Zn (*P* = 0.065). CF concentrations of iron ions in OMA patients were significantly higher than in non-OMA patients (median, 290.913 mg/L [range 191.723–36757.566 mg/L] vs. median, 24.850 mg/L [range, 11.850–57.905 mg/L]) (*r* = −0.587, *P* = 0.022). Calcium ions were not significantly different between the two groups (median, 190.722 mg/L [range, 79.417–570.046 mg/L] vs. median 80.820 mg/L [range, 77.025–132.375 mg/L]) (*r* = −0.407, *P* = 0.132).

**Table 5.** Correlation of R2 values with bio-elements (cations) in the cysts.

	Ca		Cu		Fe		Mg		Zn	
	*r*	*p*	*r*	*p*	*r*	*p*	*r*	*p*	*r*	*p*
R2, s^−1^	0.591	0.016	0.415	0.110	0.748	0.001	0.217	0.418	0.472	0.065

## Discussion

The validation study showed that OMA could be distinguished from benign and malignant ovarian cysts on the basis of CF total iron levels (sensitivity = 96.6% and specificity = 95.4%) and MR relaxometry R2 value (sensitivity = 86.2% and specificity = 70.7%). The CF iron test is a diagnostic tool with very high sensitivity and specificity. MR relaxometry is inferior to the iron test in diagnostic value, but has some advantages such as its user friendliness, non-invasiveness, and objectivity. R2 value is affected not only by the iron level but also by the contents of antioxidants and calcium ions, which may lead to a decrease in diagnostic accuracy.

First, we have long focused on iron in the cyst for the diagnosis and pathogenesis of endometriosis. To use a diagnostic test effectively in their practice, clinicians need to know how well the test distinguishes patients with OMA from those who have benign and malignant ovarian cysts. In clinical practice, imaging modalities such as TVS and MRI are suitable for diagnosing OMA [15,16]. The iron test was noninferior to TVS or MRI because it was able to distinguish between OMA and other benign and malignant ovarian cysts with 97.7% sensitivity and 96.9% specificity. At this time, however, the only way to measure iron levels is invasive or surgical. Therefore, TVS and MRI are sufficient for OMA diagnosis, and the iron test is of little significance. In the future, however, the procedure for iron measurement can be done via various imaging methods and will be noninvasive. For example, near-infrared spectroscopy can be used for measuring iron levels in the cyst in a noninvasive manner [17].

Second, for which patient is iron measured? Nagayasu et al. reported that iron levels in the cyst can predict endometriosis-related infertility [18]. High levels of iron (>326.6 mg/L) in the cyst increase the frequency of infertility [18]. On the other hand, decrease in iron levels (<64.8 mg/L) suggests malignant transformation of endometriosis [7]. These data support the idea that oxidative stress caused by iron is involved in the severity of endometriosis and the key process of carcinogenesis. It may be worthwhile to measure the iron levels to objectively predict the severity of endometriosis-related infertility in women of reproductive age and its carcinogenic potential in perimenopausal women. Since iron is a hallmark of endometriosis, quantification of CF iron levels is essential not only for diagnosing endometriosis, but also for determining treatment strategies and elucidating the pathogenesis.

Third, the validation study showed that MR relaxometry is less accurate in diagnosing OMA than iron measurements. In the training set, R2 levels were able to accurately distinguish between endometriosis and EAOC, with a cutoff value of 12.1 s^−1^ (AUC 0.899), a sensitivity of 86%, and a specificity of 94%. Since this validation study included all types of ovarian tumors, the sensitivity and specificity of R2 values for diagnosing OMA were 86.2% and 70.7%, respectively. We cannot conclude that MR relaxometry is less specific than the iron test due to the different patient populations.

Previous study has shown that the coefficient of correlation between ex vivo R2 values and working standards of purified iron has been shown to be very high (*r* = 0.994, *P* < 0.001), suggesting that paramagnetic iron directly affects the R2 value [9]. In this study, some patients showed higher R2 values compared to total iron levels, leading to reduced specificity (Figure 2). We hypothesized that the R2 value might be affected by non-iron paramagnetic molecules contained in the cyst. Therefore, we focused on antioxidants and bioelements (cations) in the cyst. The R2 value correlated well with antioxidants such as HO-1. Ovarian cysts with high HO-1 levels may have high R2 levels. Interestingly, it was found for the first time that the R2 value has a positive correlation with calcium ions in addition to paramagnetic iron. The levels of iron and calcium in the cyst are 100–1000- and 2–10-fold higher than in the blood, respectively. Iron ions were higher in OMA than non-OMA (median, 290.9 mg/L vs. 24.9 mg/L, *P* = 0.022), but calcium ions were not significantly different between the two groups (median, 190.7 mg/L vs. 80.8 mg/L, *P* = 0.132). This suggests that high calcium ions in non-OMA patients may increase R2 levels and reduce specificity. To date, there have been no reports of measuring calcium levels in the contents of ovarian cysts.

Finally, the use of MR relaxometry as a diagnostic modality has the great advantage of being a non-invasive test. In patients with OMA, an increase in R2 value may require urgent attention to avoid the progression of infertility. In addition, a decrease in R2 value can predict malignant transformation of OMA. If R2 values change over time, this information may help the clinician decide to start suitable treatment options early. In contrast, there are some open issues related to the current non-invasive approach. Further research is needed to identify paramagnetic substances such as bio-elements that affect R2 values and to improve the effectiveness of MR relaxometry.

In conclusion, the validation study revealed the following two points. Although the iron test is invasive, it has the same diagnostic performance as TVS and MRI in distinguishing between OMA and non-OMA. The diagnostic accuracy of the R2 value may be inferior to that of the iron test, probably due to the influence of paramagnetic components other than iron, but it is non-invasive and attractive.

## Data Availability

The datasets used and/or analyzed during the current study are available from the corresponding author on reasonable request.
